# Prediction of Cancer Drugs by Chemical-Chemical Interactions

**DOI:** 10.1371/journal.pone.0087791

**Published:** 2014-02-03

**Authors:** Jing Lu, Guohua Huang, Hai-Peng Li, Kai-Yan Feng, Lei Chen, Ming-Yue Zheng, Yu-Dong Cai

**Affiliations:** 1 Department of Medicinal Chemistry, School of Pharmacy, Yantai University, Yantai, Shandong, People’s Republic of China; 2 Institute of Systems Biology, Shanghai University, Shanghai, People’s Republic of China; 3 Department of Mathematics, Shaoyang University, Shaoyang, Hunan, People’s Republic of China; 4 CAS-MPG Partner Institute for Computational Biology, Shanghai Institutes for Biological Sciences, Chinese Academy of Sciences, Shanghai, People’s Republic of China; 5 Beijing Genomics Institute, Shenzhen Beishan Industrial zone, Shenzhen, People’s Republic of China; 6 College of Information Engineering, Shanghai Maritime University, Shanghai, People’s Republic of China; 7 State Key Laboratory of Drug Research, Shanghai Institute of Materia Medica, Shanghai, People’s Republic of China; University of Alberta, Canada

## Abstract

Cancer, which is a leading cause of death worldwide, places a big burden on health-care system. In this study, an order-prediction model was built to predict a series of cancer drug indications based on chemical-chemical interactions. According to the confidence scores of their interactions, the order from the most likely cancer to the least one was obtained for each query drug. The 1^st^ order prediction accuracy of the training dataset was 55.93%, evaluated by Jackknife test, while it was 55.56% and 59.09% on a validation test dataset and an independent test dataset, respectively. The proposed method outperformed a popular method based on molecular descriptors. Moreover, it was verified that some drugs were effective to the ‘wrong’ predicted indications, indicating that some ‘wrong’ drug indications were actually correct indications. Encouraged by the promising results, the method may become a useful tool to the prediction of drugs indications.

## Introduction

Cancer is the main cause of death in both developed and developing countries [1]. In 2008 alone, there were 12.7 million new cancer cases and 7.6 million cancer deaths worldwide [1]. Meanwhile, the quantity of newly approved drugs diminished continually in spite of an increase of R&D investments [2]. R&D of a drug requires comprehensive experimental testing, which often costs millions of dollars, involves several thousand animals, and takes many years to complete. However, as a result, not many chemicals have undergone the degree of testing needed to support accurate health risk assessments or meet regulatory requirements for drug approval. Thus, it is very attractive to develop quick, reliable, and non-animal-involved prediction methods, *e.g.* using structure-activity relationships (SARs), to predict the anticancer activities of chemicals.

Some pioneer studies indicated that interactive proteins are more likely to share the same functions than non-interactive ones [3], [4], [5]. Likewise, interactive compounds are also more likely to share common properties [6], [7], [8]. STITCH (Search Tool for Interactions of Chemicals, http://stitch.embl.de/) is a well-known database containing the interactions information of proteins and chemicals [9], [10]. It provides three categories of interactive compounds: (1) those participating in the same reactions; (2) those sharing similar structures or activities and (3) those with literature associations, such as binding the same target [9]. In this study, we attempted to build a prediction model of drug-indication by quantifying chemical-chemical interaction of every pair of interactive compounds. Briefly, drugs and their corresponding indications (*i.e.*, 8 kinds of cancers) were extracted from KEGG (Kyoto Encyclopedia of Genes and Genomes, http://www.genome.jp/kegg/) [11], a well-known database dealing with genomes, enzymatic pathways, and biological chemicals, and Drugbank [12], another database containing detailed information of drugs and their target information. Then, the score of each indication of the query compound was obtained from the confidence scores of interactions between the query compound and its interactive compounds using the indications of the interactive compounds. And the order from the most likely indication to the least was obtained for each drug. Finally, the prediction quality of the model was evaluated by Jackknife test and some other parameters.

In addition to build an effective prediction model, another aim of our study is to investigate the drug repositioning ability of our model. Drug repositioning, *i.e.* finding novel uses of existing drugs, is an alternative strategy towards drug development because it has the potential to speed up the process of drug approvals. Several drugs, such as thalidomide, sildenafil, bupropion and fluoxetine, have been successfully repositioned to new indications [13], [14]. Experimental approaches for drug repositioning usually employ high throughput screening (HTS) to test the libraries of drugs against biological targets of interest. More recently, several *in silico* models were developed to address the issues of drug repositioning. Iorio *et al.* predicted and validated new drug modes of action and drug repositioning from transcriptional responses [15]. Butte’s group reported two successful examples of drug repositioning based on gene expression data from diseases and drugs [16], [17]. Cheng *et al.* merged drug-based similarity inference (DBSI), target-based similarity inference (TBSI) and network-based inference (NBI) methods for drug-target association and drug repositioning [18]. In our study, according to the assumption that interactive drugs are more likely to target the same indication, we investigated the repositioning possibility of some ‘wrong’ predicted drugs by retrieving references, and attempted to propose alternative indications for some drugs.

## Materials and Methods

### Materials

The information of 98 drugs that can treat cancers was retrieved from KEGG DISEASE in KEGG [11]. These drugs can treat the following 10 kinds of cancers:

1. Cancers of the nervous system
2. Cancers of the digestive system
3. Cancers of haematopoietic and lymphoid tissues
4. Cancers of the breast and female genital organs
5. Cancers of soft tissues and bone
6. Skin cancers
7. Cancers of the urinary system and male genital organs
8. Cancers of endocrine organs
9. Head and neck cancers
10. Cancers of the lung and pleura

**Figure 1:**
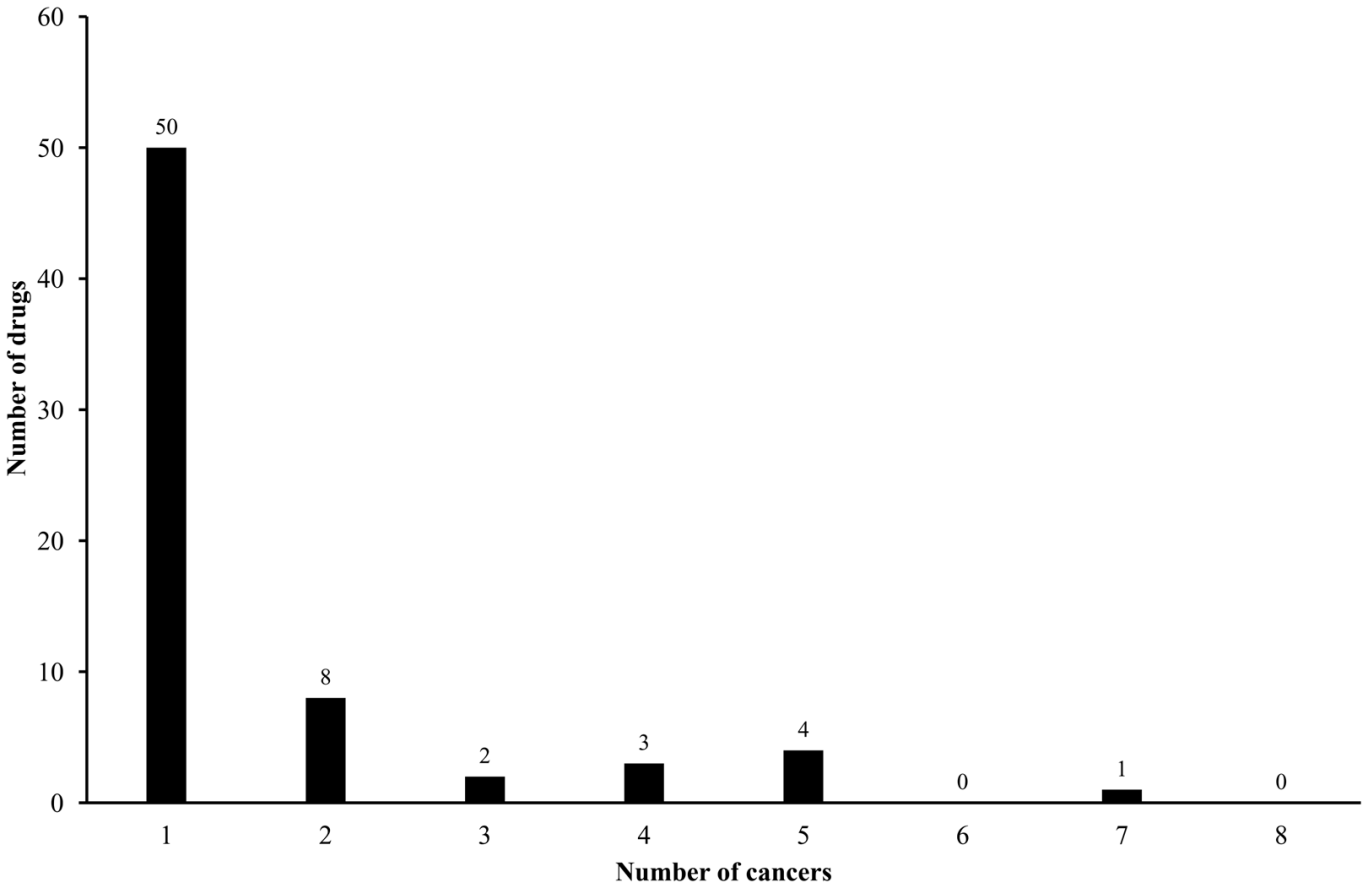
The number of drugs plotted against the number of cancers they can treat in the benchmark dataset.

Since some drugs have no information of chemical-chemical interactions, we discarded these drugs, resulting in 68 drugs. After that, we found that ‘Skin cancers’ and ‘Head and neck cancers’ only contained 3 and 4 drugs, respectively. It is not sufficient to establish an effective prediction model with only a few samples, thus these two kinds of cancers were abandoned. As a result, 68 drugs were obtained, comprising the benchmark dataset **S**. These 68 drugs were classified into 8 categories in a way that drugs that can treat one kind of cancers comprised one category. The codes of the 68 drugs and their indications can be found in **Table S1**. The number of drugs in each category is listed in column 5 of **Table 1**. For convenience, we used tags 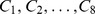 to represent each kind of cancers. Please see the column 1 and 2 of **Table 1** for the corresponding of tags and cancers. It is observed from **Table 1** that the sum of the number of drugs in each category is much larger than the different drugs in **S**, indicating that some drugs belong to more than one category, *i.e.* some drugs can treat more than one kind of cancers. In details, 50 drugs can treat only one kind of cancers, while 18 drugs can treat at least two kinds of cancers. Please refer to **Figure 1** for a plot of the number of drugs against the number of cancers they can treat. Thus, it is a multi-label classification problem which needs to assign each drug to the aforementioned 8 categories in descending order. The classifier only providing one candidate cancer that a query drug can treat is not an optimal choice. Similar to the situation when dealing with proteins and compounds with multiple attributions [7], [19], the proposed method also needs to provide a series of candidate cancers, ranging from the most likely cancer to the least likely one.

**Table 1:** The number of drugs in each category of **S**_tr_, **S**_te_, **S** and **S**_ite_.

Tag	Cancer	Number of drugs			
		Trainingdataset S_tr_	Validation testdataset S_te_	Total in S	Independenttest dataset S_ite_
*C*_1_	Cancers of the nervous system	8	1	9	1
*C*_2_	Cancers of the digestive system	8	5	13	6
*C*_3_	Cancers of haematopoietic andlymphoid tissues	24	6	30	21
*C*_4_	Cancers of the breast and femalegenital organs	13	6	19	11
*C*_5_	Cancers of soft tissues and bone	4	6	10	2
*C*_6_	Cancers of the urinary system andmale genital organs	9	5	14	9
*C*_7_	Cancers of endocrine organs	5	2	7	1
*C*_8_	Cancers of the lung and pleura	6	3	9	7
*–*	Total	77	34	111	58

To better evaluate the proposed method, the benchmark dataset **S** was divided into one training dataset **S_tr_** and one validation test dataset **S**_te_, *i.e.* **S** = **S**_tr_∪**S**_te_ and **S**_tr_∩**S**_te_ = Ø, where drugs that can only treat exact one kind of cancer and half of drugs that can treat at least two kinds of cancers comprised **S**_tr_, while **S**_te_ contained the rest drugs in **S**. The number of drugs in each category for **S**_tr_ and **S**_te_ is listed in column 3 and 4 of **Table 1**, respectively.

In addition, to test the generalization of the proposed method, we extracted 59 drug compounds from Drugbank [12], which are not in the benchmark dataset *S*. After excluding drug compounds without information of chemical-chemical interactions, 44 drugs were obtained, comprising the independent test dataset **S**_ite_. The number of drugs in each category of **S**_ite_ is listed in column 6 of **Table 1** and the detailed information of these drug compounds including their codes and indications can be found in **Table S2**.

### Chemical-chemical Interactions

In recent years, the information of chemical-chemical interactions is penetrating into the prediction of various attributions of compounds [7], [8], [20]. The basic idea is that interactive compounds are more likely to share common functions than non-interactive ones. Compared with the information based on chemical structure, it includes other essential properties of compounds, such as compounds activities, reactions, and so on.

The information of interactive compounds was downloaded from STITCH (chemical_chemical.links.detailed.v3.1.tsv.gz) [9]. In the obtained file, each interaction consists of two compounds and five kinds of scores entitled ‘Similarity’, ‘Experimental’, ‘Database’, ‘Textmining’ and ‘Combined_score’. In details, the first four kinds of scores are calculated based on the compound structures, activities, reactions, and co-occurrence in literature, respectively, while the last kind of score ‘Combined_score’ integrates the aforementioned four scores. Thus, it is used in this study to indicate the interactivity of two compounds, *i.e.* two compounds are interactive compounds if and only if the ‘combined_score’ of the interaction between them is greater than zero. In fact, the value of ‘combined_score’ also indicates the strength of the interaction, *i.e.* the likelihood of the interaction’s occurrence. Thus, it is termed as confidence score in this study. For convenience, we denote the confidence score of the interaction between *c*_1_ and *c*_2_ by 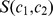. In particular, if *c*_1_ and *c*_2_ are non-interactive compounds, 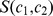 is set to zero.

112 drug compounds were investigated in this study as described in Section “Materials”, and 1,393 chemical-chemical interactions whose confidence scores were greater than zero were obtained. Among the interactions which scores are greater than zero, 50 of them belonged to the label ‘Similarity’, 4 belonged to ‘Experiment’, 114 belonged to ‘Database’, and 1,352 belonged to ‘Textmining’. It is necessary to point out that some drug interactions had two or more than two kinds of scores. As far as the quantity of chemical-chemical interactions is concerned, the tag ‘Textmining’ contributed most to the construction of the prediction method described in Section “The method based on chemical-chemical interactions”.

### Prediction Method

#### The method based on chemical-chemical interactions

Systems biology has been applied extensively into the predictions of properties of proteins and compounds and is deemed to be more efficient than some conventional methods [7], [20], [21], [22]. In this study, we attempt to classify cancer drugs into the aforementioned 8 categories based on chemical interactions.

Suppose there are *n* drugs in the training set 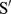, say 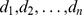. Cancers that 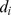
can treat is represented as follows:
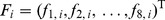
(1)where **T** is the transpose operator and



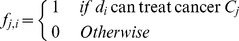
(2)For a query drug 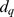, which cancer it can treat can be determined by its interactive compounds in 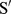. To evaluate the likelihood that 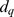 can treat cancer 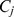, we calculated a score as follows:
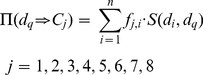
(3)


Larger score of 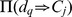 indicates that it is more likely the query drug can treat cancer 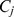. And 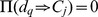 suggests that the probability that the query drug can treat cancer 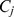 is zero, because there are no interactive compounds in 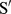 that can treat cancer 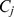.

As mentioned in Section “Materials”, predicting which cancers a drug can treat is a multi-label classification problem. A reliable classifier should provide not only the most likely cancer but also a series of candidate cancers, ranging from the most likely one to the least likely one. According to the results of **Eq. 3**, it is easy to arrange the candidate cancers using the decreasing order of the corresponding scores. For example, if the results of **Eq. 3** are:
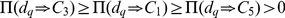
(4)


it means that there are three candidate cancers of 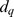, where the most likely cancer it can treat is 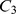, followed by 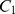 and 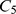. Furthermore, 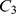 is called the 1^st^ order prediction, and 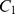 is the 2^nd^ order prediction, and so forth.

### The Method Based on Molecular Descriptors

To compare our method with other methods, the method based on molecular descriptors was constructed as follows. The structure optimization of each drug compound was performed using the AM1 semi-empirical method implemented in AMPAC 8.16 [23]. 454 descriptors including constitutional, topological, geometrical, electrostatic, and quantum-chemical descriptors were calculated by Codessa 2.7.2 [24]. To encode each drug compound effectively, the descriptors with missing values were discarded, resulting in 355 descriptors, *i.e.* each drug compound *d* can be represented by a 355-D (dimension) vector which can be formulated as follows:
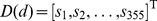
(5)where **T** is the transpose operator. Accordingly, the relationship of two drugs *d*_1_ and *d*_2_ can be calculated by the following formula:
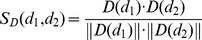
(6)where 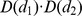 is the dot product of 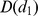 and 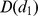, while 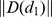 and 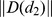 is the modulus of 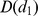 and 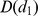, respectively.

Similar to the method based on chemical-chemical interactions, the score that a query drug 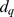 can treat cancer 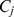 can be calculated by the following formula:
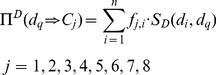
(7)


The rest procedure is the same as that of the method based on chemical-chemical interactions, which also provides a series of candidate cancers that 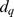 can treat, ranging from the most likely one to the least one.

### Validation and Evaluation

Jackknife test is one of the most popular methods for evaluating the performance of classifiers. During the test, each sample is singled out one-by-one and predicted by the classifier trained by the rest samples in the dataset. The test procedure is open, thereby avoiding arbitrary problem [7]. Therefore, the outcome obtained by Jackknife test is always unique for a given dataset. In view of this, many investigators have adopted it to evaluate the accuracies of their classifiers in recent years [25], [26], [27], [28], [29].

As described in Section “Prediction method”, the methods in this study can provide a series of candidate cancers for a given query drug. The *j*-th order prediction accuracy is computed by the following formula [7], [8]:
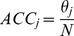
(8)where *N* is the total number of drugs in the dataset and 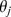 is the number of drugs such that their *j*-th predictions are the true cancers that they can treat. It is obvious that 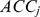 measures the quality of the *j*-th order prediction. If the true cancers that a query drug can treat are positioned in low order, it is deemed as an optimal predicted result. Thus, high 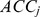 with low order number *j* and low 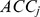 with high order number *j* indicate a good performance of the classifier. 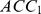 is the most important indicator of the performance of the classifier.

To evaluate the methods more thoroughly, we calculated the prediction accuracy on cancer 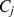 for the *i*-th order prediction as follows:
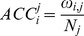
(9)where *N_j_* is the number of drugs that can treat cancer 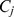 in the dataset and 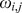 is the number of drugs such that its *i*-th order prediction is correctly predicted to treating cancer 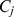.

In addition, another measurement was taken, which was adopted in some previous studies [6], [7], [8] and can be calculated as follows:
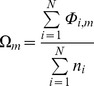
(10)where *m* represents the first *m* predictions that are taken into consideration, 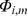 is the number of the correct predictions of the *i-*th drug compound among its first *m* predictions, *n_i_* is the number of cancers that the *i-*th drug compound can treat. It is easy to deduce that 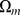 means the proportion of all true cancers that the samples in the dataset can treat covered by the first *m* predictions of each sample in it. It can be seen from **Figure 1** that different drug compounds may have different numbers of cancers they can treat. In view of this, the parameter *m* in **Eq. 10** usually takes the value of the smallest but no less than the average number of cancers that drug compounds in the dataset can treat. It can be computed by



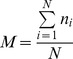
(11)Generally speaking, higher 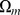 suggests better performance of the method.

## Results and Discussion

As described in Section “Materials”, the benchmark dataset **S** was divided into a training dataset **S**_tr_ and a validation test dataset **S**_te_, which contained 59 and 9 drugs, respectively. In addition, an independent test dataset **S_ite_** containing 44 drugs was constructed to test the generalization of the method. The predicted method introduced in Section “The method based on chemical-chemical interactions” was used to make prediction. The detailed predicted results are given as follows.

### Performance of the Method Based on Chemical-chemical Interactions on the Training Dataset

As for the 59 drugs in the training dataset **S**_tr_, the predictor was performed and evaluated by Jackknife test. Listed in column 2 of **Table 2** are the 8 prediction accuracies calculated by **Eq. 8**, from which we can see that the 1^st^ order prediction accuracy was 55.93%, while the 2^nd^ order prediction accuracy was 22.73%. It is also observed from column 2 of **Table 2** that the prediction accuracies generally followed a descending trend with the increase of the order number, indicating that the proposed method arranged the candidate cancers in the training dataset quite well. In details, for each order prediction, we calculated the accuracies of each kind of cancer according to **Eq. 9**, which were listed in row 2–9 of **Table 3**. It can be seen that most of the 0.00% accuracy occurred when the prediction order was high, indicating that for each kind of cancer, it was better predicted with lower order number of the predictions. The average number of cancers which drugs in **S**_tr_ can treat was 1.31 (77/59), calculated by **Eq. 11**. It means that the average success rate would be only 16.38% if ones make prediction by random guesses, *i.e.* randomly assign a cancer indication to each sample, which is much lower than the 1^st^ order prediction accuracy obtained by our method. Because the average number of cancers a drug can treat is 1.31, the first 2 order predictions of each sample in **S**_tr_ were taken to calculate the proportion of true cancers that samples in **S**_tr_ can treat covered by these predictions according to **Eq. 10**, obtaining a ratio of 61.04%.

**Table 2:** Prediction accuracies of the method based on chemical-chemical interactions on **S**_tr_ **, S**_te_ and **S**_ite._

Prediction order	S_tr_	S_te_	S_ite_
1	55.93%	55.56%	59.09%
2	22.73%	66.67%	29.55%
3	20.34%	44.44%	6.82%
4	8.47%	66.67%	11.36%
5	5.08%	22.22%	6.82%
6	10.17%	55.56%	2.27%
7	6.78%	55.56%	13.64%
8	0.00%	11.11%	2.27%

**Table 3:** Prediction accuracies on 8 kinds of cancers for each order prediction obtained by our predictor.

Dataset	Prediction order	*C*_1_	*C*_2_	*C*_3_	*C*_4_	*C*_5_	*C*_6_	*C*_7_	*C*_8_
	1	0.00%	25.00%	95.83%	46.15%	0.00%	0.00%	40.00%	0.00%
	2	37.50%	25.00%	4.17%	53.85%	0.00%	11.11%	0.00%	0.00%
	3	62.50%	37.50%	0.00%	0.00%	0.00%	22.22%	20.00%	16.67%
**S**_tr_	4	0.00%	12.50%	0.00%	0.00%	0.00%	22.22%	0.00%	33.33%
	5	0.00%	0.00%	0.00%	0.00%	0.00%	0.00%	0.00%	50.00%
	6	0.00%	0.00%	0.00%	0.00%	50.00%	44.44%	0.00%	0.00%
	7	0.00%	0.00%	0.00%	0.00%	50.00%	0.00%	40.00%	0.00%
	8	0.00%	0.00%	0.00%	0.00%	0.00%	0.00%	0.00%	0.00%
	1	0.00%	0.00%	83.33%	0.00%	0.00%	0.00%	0.00%	0.00%
	2	0.00%	0.00%	16.67%	83.33%	0.00%	0.00%	0.00%	0.00%
	3	100.00%	0.00%	0.00%	16.67%	0.00%	0.00%	50.00%	33.33%
**S**_te_	4	0.00%	80.00%	0.00%	0.00%	33.33%	0.00%	0.00%	0.00%
	5	0.00%	0.00%	0.00%	0.00%	16.67%	0.00%	0.00%	33.33%
	6	0.00%	0.00%	0.00%	0.00%	16.67%	80.00%	0.00%	0.00%
	7	0.00%	20.00%	0.00%	0.00%	33.33%	20.00%	0.00%	33.33%
	8	0.00%	0.00%	0.00%	0.00%	0.00%	0.00%	50.00%	0.00%
	1	0.00%	50.00%	66.67%	36.36%	0.00%	33.33%	100.00%	14.29%
	2	100.00%	16.67%	14.29%	36.36%	0.00%	11.11%	0.00%	42.86%
	3	0.00%	16.67%	0.00%	18.18%	0.00%	0.00%	0.00%	0.00%
**S**_ite_	4	0.00%	0.00%	9.52%	9.09%	0.00%	22.22%	0.00%	0.00%
	5	0.00%	16.67%	4.76%	0.00%	0.00%	0.00%	0.00%	14.29%
	6	0.00%	0.00%	0.00%	0.00%	50.00%	0.00%	0.00%	0.00%
	7	0.00%	0.00%	4.76%	0.00%	50.00%	33.33%	0.00%	14.29%
	8	0.00%	0.00%	0.00%	0.00%	0.00%	0.00%	0.00%	14.29%

### Performance of the Method Based on Chemical-chemical Interactions on the Validation Test Dataset

As for the 9 drugs in the validation test dataset **S**_te_, their candidate cancers were predicted by the method described in Section “The method based on chemical-chemical interactions” based on the information of the drugs in **S**_tr_. 8 prediction accuracies calculated by **Eq. 8** were listed in column 3 of **Table 2**. It can be seen that the 1^st^ order prediction accuracy was 55.56%, while the 2^nd^ order one was 66.67%. It is also observed from **Table 2** that the prediction accuracies of this dataset were generally higher than those of the training dataset, due to the fact that drugs in **S**_te_ can treat two or more than two kinds of cancers, while most drugs in **S**_tr_ can only treat one kind of cancers. Similarly, we calculated the accuracies of each kind of cancer for the 1^st^, 2^nd^, …, 8^th^ order prediction by **Eq. 9**. Row 10–17 of **Table 3** listed them. The average number of cancers that drugs in **S**_te_ can treat was 3.78 (34/9), indicating that if ones make prediction by random guesses, the average success rate would be 47.22%, which is significantly lower than the 1^st^ and 2^nd^ order accuracies listed in column 3 of **Table 2**. This suggests that the performance of the method on the validation test dataset is fairly good. Since the average number of cancers that drugs in **S**_te_ can treat was 3.78, the first 4 order predictions of each sample in **S**_te_ were considered. According to **Eq. 10**, 61.76% of true cancers were correctly predicted by the first 4 order predictions.

### Performance of the Method Based on Chemical-chemical Interactions on the Independent Test Dataset

The candidate cancers of the 44 drugs in the independent test dataset **S**_ite_ were also predicted by our predictor based on the drug information in **S**_tr_. 8 prediction accuracies were obtained and listed in column 4 of **Table 2**, from which we can see that the 1^st^ order prediction accuracy was 59.09%, while the 2^nd^ order prediction accuracy was 29.55%. To better evaluate the method, the prediction accuracies on each kind of cancer for the 8 order predictions were calculated by **Eq. 9** and listed in row 18–25 in **Table 3**. The average number of cancers that drugs in **S**_ite_ can treat was 1.32 (58/44), suggesting that if ones make prediction by random guesses, the average success rate would be 16.5%, which is much lower than the 1^st^ order prediction accuracy obtained by our method. Because the average number of drug indications was 1.32, the first 2 order prediction of each sample in **S**_ite_ was considered. According to **Eq. 10**, 67.24% of true cancers were correctly predicted by the first 2 order predictions.

### Comparison with other Methods

To indicate the effectiveness of our method for the prediction of drugs cancer indications, some other methods were built to make comparison.

The method based on molecular descriptors described in Section “The method based on molecular descriptors” was conducted on **S**_tr_ with its performance evaluated by Jackknife test. The 8 prediction accuracies calculated by **Eq. 8** were listed in column 2 of **Table 4**, from which we can see that the 1^st^ order prediction accuracy was 41.38%. It is much lower than the 1^st^ order prediction accuracy of 55.93% obtained by the method based on chemical-chemical interactions. Also, for drugs in **S**_te_ and **S**_ite_, their cancer indications were predicted by molecular descriptors on **S**_tr_. The prediction accuracies were listed in column 3 and 4 in **Table 4**. In details, the 1^st^ order prediction accuracy on **S**_te_ and **S**_ite_ were 55.56% and 44.19%, respectively. Compared with the prediction accuracies of 55.56% on **S**_te_ and 59.09% on **S**_ite_ using chemical interactions, they performed at the same level on **S**_te_, and chemical interactions are much better than chemical descriptors on **S**_ite_. In addition, we considered the first 2-order, 4-order and 2-order predictions on **S**_tr_, **S**_te_, and **S**_ite_ due to the average number of cancers that drugs in these datasets can treat. The proportion of true cancers that samples in **S**_tr_, **S**_te_, and **S**_ite_ can treat covered by these predictions were 51.39%, 58.82% and 49.12%, respectively, which were all lower than the corresponding proportions of 61.04%, 61.76% and 67.24%, respectively, obtained by the method based on chemical-chemical interactions. Therefore, the method based on chemical interactions was superior to the method based on molecular descriptors.

**Table 4:** Prediction accuracies of the method based on molecular descriptors on **S**_tr_, **S**_te_ and **S**_ite._

Prediction order	S_tr_	S_te_	S_ite_
1	41.38%	55.56%	44.19%
2	22.41%	77.78%	20.93%
3	18.97%	55.56%	18.60%
4	6.90%	33.33%	13.95%
5	8.62%	33.33%	11.63%
6	6.90%	33.33%	9.30%
7	5.17%	55.56%	11.63%
8	13.79%	33.33%	2.33%

As was described in the above three sections, the performance of our method was much better than that of the random guesses, which randomly assigned a cancer indication to a query drug. Here, another random guesses method was applied to evaluate our method from a different aspect. For any query drug *d_q_*, we randomly selected a drug compound in the training set, say *d*, and assigned true cancers that *d* can treat to *d_q_*, *i.e.* the predicted cancers of *d_q_* were same as the true cancers that *d* can treat. Since there is no order information in the predicted candidate cancers for each sample, the measures provided by Section “Validation and evaluation” cannot evaluate the performance of this method. Thus, Recall and Precision [30], [31] were employed to evaluate its performance, which can be computed by.
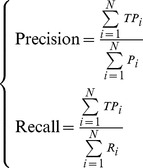
(12)where 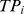 is the number of correct predicted cancers for the *i*-th drug compound, 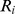 represents the numbers of cancers which the *i*-th drug compound can treat, 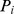 represents the numbers of predicted cancers for the *i*-th drug compound, and *N* is total number of tested samples.

The random guess method described in the above paragraph was conducted on **S**_tr_ with its performance evaluated by Jackknife test. The Precision and Recall were 15.29% and 16.88%, respectively. For the predicted results on **S**_tr_ by chemical-chemical interactions, the 1^st^ order prediction of each sample were picked, obtaining Precision of 55.93% and Recall of 42.86%, which were much higher than the random guess method.

It is easy to see that our method depend deeply on the confidence scores of chemical-chemical interactions. To test the importance of these scores, we randomly exchanged the confidence scores of some interactions. Based on the random permutations, the data were evaluated by Jackknife test on the training dataset **S**_tr_. The 1^st^ order prediction accuracy was 23.73%, while the other prediction accuracies of 2^nd^, 3^rd^,…,8^th^ order prediction were 18.64%, 11.86%, 18.64%, 20.34%, 15.25%, 13.56%, 8.47%, respectively. It is observed that the 1^st^ order prediction accuracy obtained by random permutation was much lower than the 55.93% obtained by chemical interactions. Furthermore, the 8 prediction accuracies were not followed a descending trend with the increase of the order number, indicating that the candidate cancers were not arranged well. This implicates that confidence scores are very important to the predictions.

## Discussion

26 1^st^ order predictions were ‘wrong’ in the training dataset, that is, the predicted cancer indications of these drugs were not recorded in KEGG. These 26 drugs and their 1^st^ order predictions were available in **Table S3**. However, some references reported that 23 of these 26 drugs were actually effective to their ‘wrong’ indications, and it was the same with 3 of the 4 drugs in the validation test dataset (See **Table S3** for the detailed 4 drugs and their 1^st^ order prediction) and 13 of the 18 drugs in the independent test dataset (See **Table S3** for detailed 18 drugs and their 1^st^ order prediction). Thus, we hope that our prediction model can provide some information of drug repositioning. In the following paragraphs, we cited some references to support our predicted results.

### Twenty-three Wrong Predicted Pairs of Drug and Indication in the Training Dataset

#### Cisplatin-Cancers of haematopoietic and lymphoid tissues

Cisplatin (KEGG ID: D00275), “penicillin of cancer drugs”, is widely prescribed for many cancer treatments, such as testicular, ovarian, bladder, lung, stomach cancers, and lymphoma [32], [33], [34]. Prasad *et al.* investigated the effect of cisplatin on the Dalton’s lymphoma, and concluded that cisplatin can induce complete regression of ascites Dalton’s lymphoma in mice [35].

#### Ifosfamide-Cancers of haematopoietic and lymphoid tissues

Ifosfamide (D00343) can be used to treat germ cell testicular cancer, cervical cancer, small cell lung cancer, non-Hodgkin’s lymphoma, and so on [36]. Extranodal natural killer/T-cell lymphoma, nasal type (ENKL) is Epstein-Barr virus-associated lymphoid malignancies, and patients with stage IV, relapsed or refractory ENKL have dismal prognoses. Yamaguchi *et al.* explored a new regimen SMILE, including the steroid dexamethasone, methotrexate, ifosfamide, L-asparaginase, and etoposide, and concluded that SMILE was effective for this kind of disease [37], [38].

#### Lomustine-Cancers of haematopoietic and lymphoid tissues

Lomustine (D00363) is a component of the combination chemotherapy for treating primary and metastatic brain tumors, and also used as a secondary therapy for refractory or relapsed Hodgkin’s disease [39]. Moreover, previous studies reported that lomustine can be considered for the treatment of canine lymphoma in dogs [40], [41], [42], [43], although it induced common but not life-threatening toxicity [44].

#### Mitotane-Cancers of the urinary system and male genital organs

Mitotane (D00420) is the first-line drug for metastatic adrenocortical carcinoma [45], [46], [47], and also used for the adjuvant therapy after removing the primary tumor [48]. However, mitotane treatment can induce some side effects, such as adrenal insufficiency and male hypogonadism [49].

#### Procarbazine-Cancers of haematopoietic and lymphoid tissues

Procarbazine (D00478) is used to treat human leukemias [50]. MOPP (mechlorethamine, oncovin, procarbazine, and prednisone) is the first combination chemotherapy regimen for treating Hodgkin lymphoma (HL) [51]. And BACOPP regimen (bleomycin, adriamycin, cyclophosphamide, vincristine, procarbazine, and prednisone) improved both tolerability and efficacy of older HLs, although it induced a high rate of toxic deaths [52].

#### Temozolomide-Cancers of haematopoietic and lymphoid tissues

Temozolomide (D06067) is an oral alkylating agent used for the treatment of anaplastic astrocytoma and glioblastoma multiforme [53]. Reni *et al.* reported that temozolomide was effective for immunocompetent patients with recurrent primary brain lymphoma, and its toxicity was negligible [54].

#### Thiotepa-Cancers of haematopoietic and lymphoid tissues

Thiotepa (D00583) is an alkylating agent to treat breast, ovarian, and bladder cancer [55]. A regimen of reduced-intensity conditioning with thioteopa, fludarabine, and melphalan produced remissions and a limited transplant mortality rate in most multiple myeloma patients [56]. Moreover, Kolb *et al.* studied a phase II nonrandomized single-arm trial using TVTG regimen (topotecan, vinorelbine, thiotepa, dexamethasone, and gemcitabine) for relapsed or refractory leukemia, and reported 47% response rate of patients and acceptable toxicities [57].

#### Floxuridine-Cancers of the digestive system

Floxuridine (D04197) is used to treat hepatic metastases of gastrointestinal adenocarcinomas, and also used for palliation of cancers in the liver and gastrointestinal tract [58]. Moreover, hepatic arterial infusion (HAI) can significantly enhance the antitumor activity of floxuridine against colorectal liver metastases, as compared with systemic infusion [59].

#### Carboplatin-Cancers of haematopoietic and lymphoid tissues

Carboplatin (D01363) is approved with less side effects compared with its parent compound cisplatin in the clinical treatment, and mainly used to treat ovarian, lung, head cancers, and so on [34]. Through a phase II trial, Gopal *et al.* reported that GCD (gemcitabine, carboplatin, dexamethasone, and rituximab) was a safe and effective outpatient salvage regimen for relapsed lymphoma [60]. And Moskowitz *et al.* also reported that ICE regimen (ifosfamide, carboplatin, and etoposide) was effective for patients with non-Hodgkin’s lymphoma [61].

#### Epirubicin-Cancers of haematopoietic and lymphoid tissues

Epirubicin (D02214) is a component of adjuvant therapy in patients after resection of the primary breast cancer [62]. When used to treat chronic lymphocytic leukaemia, the combination of fludarabine and epirubicin achieved a higher response rate and a more rapid response, as compared with fludarabine alone [63].

#### Gemcitabine- Cancers of haematopoietic and lymphoid tissues

Gemcitabine (D01155) is a nucleoside analog that can treat breast, non-small cell lung, and pancreatic cancer [64]. Moreover, a regimen including gemcitabine, carboplatin, dexamethasone, and rituximab was reported to be effective for relapsed lymphoma [60].

#### Vinorelbine-Cancers of the breast and female genital organs

Vinorelbine (D01935) is used to treat non-small cell lung cancer [65]. Aapro *et al.* explored the effects of vinorelbine on metastatic breast cancer (MBC), and concluded that oral vinorelbine was highly effective and well tolerated for patients with MBC, no matter a single-agent or in combination with other agents [66]. Moreover, vinorelbine was also considered as a promising alternative for older patients with advanced breast cancers because of its clinical activity and low side effects [67].

#### Irinotecan-Cancers of the breast and female genital organs

Irinotecan (D01061) is used to treat metastatic colorectal cancer and extensive small cell lung cancer [68]. Previous studies reported that irinotecan was effective for the refractory metastatic breast cancer after anthracyclines or taxanes treatment [69], [70]. Moreover, the combination of irinotecan and docetaxel also achieved a high response rate in pre-treated advanced breast cancer patients [71].

#### Capecitabine-Cancers of the breast and female genital organs

Capecitabine (D01223) is an oral agent used for the treatment of metastatic breast cancers, and toxicities are generally manageable [72], [73], [74].

#### Gefitinib-Cancers of the breast and female genital organs

Gefitinib (D01977) is used for the continued treatment of patients with locally advanced or metastatic non-small cell lung cancer after failure of either platinum-based or docetaxel chemotherapies [75]. Moreover, gefitinib is the first selective inhibitor of the epidermal growth factor receptor (EGFR) tyrosine kinase, which controls cell proliferation by activating the Ras signal transduction cascade [75]. Thus, gefitinib may be a promising agent used for the treatment of metaplastic breast carcinoma with frequent expresses of EGFR [76].

#### Sorafenib-Cancers of the lung and pleura

Sorafenib (D06272) is a multi-kinase inhibitor by targeting Raf/MEK/ER pathway, and approved for the treatment of advanced renal cell carcinoma and advanced hepatocellular carcinoma [77]. Blumenschein *et al.* reported that continuous treatment with sorafenib 400 mg twice daily helped disease stabilization of patients with advanced non-small-cell-lung cancer, which is associated with Raf/MEK/ER [78].

#### Paclitaxel-Cancers of the lung and pleura

Paclitaxel (D05333) is used for the treatment of Kaposi’s sarcoma, lung cancer, ovarian cancer, and breast cancer [79]. Hensing *et al.* explored the effects of carboplatin and paclitaxel (C/P) on elderly patients with advanced non-small-cell-lung cancer, as compared with younger patients. The study indicated that the survival rates and quality-of-life of elderly and young groups are not different, so C/P should be a reasonable regimen for elderly patients with this kind of cancer [80].

#### Dacarbazine-Cancers of the breast and female genital organs

Dacarbazine (D00288) is used to treat metastatic malignant melanoma and Hodgkin’s disease [81]. Moreover, the regimen including cisplatin, adriamycin, and dacarbazine was reported to be effective for patients with metastatic uterine and ovarian mixed mesodermal sarcomas [82].

#### Sunitinib-Cancers of the breast and female genital organs

Sunitinib (D06402) is an approved drug for the treatment of renal cell carcinoma and imatinib-resistant gastrointestinal stromal tumor [83]. Moreover, previous study reported that single-agent sunitinib achieved objective response rate of 11% in MBC [84], and the combination of sunitinib and paclitaxel was also well tolerated in patients with locally advanced or MBC [85].

#### Flutamide-Cancers of the breast and female genital organs

Flutamide (D00586) is an antiandrogen for the management of prostate carcinoma [86]. Dimonaco *et al.* reported that flutamide had an inhibitory effect on the growth of rat breast cancer [87].

#### Leucovorin-Cancers of the breast and female genital organs

Leucovorin (D01211) is used to treat osteosarcoma after high-dose methotrexate therapy [88]. Moreover, a phase II study showed that the regimen of weekly mitoxantrone, 5-fluorouracil, and leucovorin (MFL) was well tolerated and moderately effective to treat MBC [89]. And a phase 3 trial of eniluracil +5-fluorouracil+leucovorin in MBC is also ongoing [90].

#### Goserelin-Cancers of the breast and female genital organs

Goserelin (D00573) is a luteinizing hormone blocker, and reduces the oestrogen level. Thus, goserelin can improve the long-term survival of premenopausal women with early breast cancer [91].

#### Fluorouracil-Cancers of haematopoietic and lymphoid tissues

Fluorouracil (5-FU, D00584) is used to treat multiple actinic and solar keratoses [92]. Takeno *et al.* reported that a case with advanced esophageal cancer accompanying multiple lymph node metastases was successfully treated by the combination of docetaxel, cisplatin, and fluorouracil [93].

### Three Wrong Predicted Pairs of Drug and Indication in the Validation Test Dataset

#### Dactinomycin-Cancers of haematopoietic and lymphoid tissues

Dactinomycin (D00214) is an antineoplastic agent, which can treat Wilms’ tumor and rhabdomyosarcoma [94]. However, it is reasonable to assume this compound for the treatment of cancers of lymphoid tissues because it induced the tumor regression of childhood lymphoma [95].

#### Mitomycin-Cancers of haematopoietic and lymphoid tissues

Mitomycin (D00208) is an chemotherapy drug for treating cancers of lip, oral cavity, digestive organ, and so on [96]. Mitomycin treated a case with localized conjunctival mucosa-associated lymphoid tissue lymphoma, and had minimal local controllable side effects [97]. Moreover, mitomycin was about 5 times more potent than porfiromycin (methyl mitomycin) when inhibiting the tumor growth in the lymphoma L1210 [98], but M-83 (7-N-(p-hydroxyphenyl)mitomycin) showed significantly higher therapeutic activity than mitomycin in lymphoma EL4 [99].

#### Etoposide-Cancers of the breast and female genital organs

Etoposide (D04107) is used to treat refractory testicular tumors, small cell lung cancer, lymphoma, non-lymphocytic leukemia, glioblastoma multiforme, and so on [100]. Poplin *et al.* reported that oral etoposide had a modest activity for chemonaive patients with metastatic endometrial cancer, but the minimal toxicity of this drug made it possible for the combination chemotherapy [101]. Moreover, etoposide was reported to be one of the most effective agents for trophoblastic disease [102], and the combination of etoposide, ifosfamide/mesna, and cisplatin (VIP) appeared to be active in advanced cervical cancer [103].

### Thirteen Wrong Predicted Pairs of Drug and Indication in the Independent Test Dataset

#### Diethylstilbestrol-Cancers of the breast and female genital organs

Diethylstilbestrol (DrugBank ID: DB00255) is used for the treatment of prostate cancer [104]. Moreover, Peethambaram *et al.* reported that diethylstilbestrol was more effective than tamoxifen in postmenopausal women with MBC, but this treatment was usually associated with toxicity such as nausea, edema, vaginal bleeding, and cardiac problems [105].

#### Bleomycin-Cancers of the nervous system

Bleomycin (DB00290) is a drug for the palliative treatment of malignant neoplasm, such as lung cancers and lymphomas [106]. Moreover, Takeuchi *et al.* reported that bleomycin was effective for the patients with gliomas, and the response rate was more than 50% [107]. And electrochemotherapy enhanced bleomycin uptake and achieved 69% complete elimination of glial cell derived tumor cells [108].

#### Bexarotene-Cancers of the lung and pleura

Bexarotene (DB00307) is used orally to treat skin manifestations of cutaneous T-cell lymphoma in patients after at least one prior systemic therapy [109]. Moreover, bexarotene was effective for preventing the growth and progression of lung tumor in mice [110], and the combination of bexarotene+paclitaxel or bexarotene+vinorelbine had significantly greater antitumor effects than the single agent [111].

#### Dexrazoxane-Cancers of haematopoietic and lymphoid tissues

Dexrazoxane (DB00380) can reduce the incidence and severity of cardiomyopathy associated with doxorubicin administration in women with MBC [112]. Moreover, dexrazoxane was used as a cardioprotective agent that can attenuate the QT and QTc dispersion associated with epirubicin-based chemotherapy in patients with aggressive non-Hodgkin lymphoma [113], and prevent or reduce cardiac injury associated with doxorubicin administration for childhood acute lymphoblastic leukemia [114], [115].

#### Valrubicin-Cancers of haematopoietic and lymphoid tissues

Valrubicin (DB00385) is used to treat bladder cancer [116]. Moreover, valrubicin was reported to inhibit the growth of leukemia cells [117], [118].

#### Zoledronate-Cancers of the breast and female genital organs

Zoledronate (DB00399) is used for the treatment of patients with multiple myeloma and bone metastases from solid tumors when combining standard antitumor therapy [119]. Moreover, Steinman *et al.* reported that zoledronate increased disease-free survival in postmenopausal and in premenopausal, hormone-suppressed breast cancer patients, but had no antitumor effect for premenopausal patients without ovarian suppression [120].

#### Pemetrexed-Cancers of the digestive system

Pemetrexed (DB00642) is used as a single agent to treat locally advanced or metastatic NSCLC after a prior chemotherapy, and also used for the treatment of adults’ malignant pleural mesothelioma in combination with cisplatin [121]. A phase II study reported that pemetrexed disodium was effective for patients with advanced gastric cancer, and the supplementation of folic acid decreased the toxicity with no compromise in efficacy [122].

#### Fluoxymesterone-Cancers of haematopoietic and lymphoid tissues

Fluoxymesterone (DB01185) is used for the palliative treatment of androgenresponsive recurrent mammary cancer in postmenopausal women with more than one year but less than five years [123]. Moreover, Bai *et al.* reported that fluoxymesterone stimulated the proliferation and differentiation of normal erythropoietic burst-forming units that are affected by inhibitory factors produced by leukemic cells [124].

#### Genistein-Cancers of the lung and pleura

Genistein (DB01645) is an experimental agent for the treatment of prostate cancer [125]. Moreover, Lian *et al.* reported that genistein may be a promising agent to treat NSCLC because genistein induced apoptosis of NSCLC cells by a p53-independent pathway [126].

#### Vorinostat-Cancers of the urinary system and male genital organs

Vorinostat (DB02546) is used to treat skin manifestations of cutaneous T-cell lymphoma patients with progressive, persistent or recurrent disease on or after two systemic therapies [127]. Pratap *et al.* reported that vorinostat inhibited tumor growth and associated osteolysis in the prostate cancer cells, but increased normal bone loss [128].

#### Ixabepilone-Cancers of the digestive system

Ixabepilone (DB04845) is investigated for the treatment of breast cancer, head and neck cancer, lung cancer, and so on [129]. Moreover, ixabepilone was reported to be active against advanced or metastatic gastric cancers [130], [131].

#### Trabectedin-Cancers of the lung and pleura

Trabectedin (DB05109) is used to treat soft tissue sarcoma and ovarian cancer, and also investigated for the treatment of gastric cancer, and so on [132]. Moreover, Massuti *et al.* reported that trabectedin had modest activity in NSCLC patients pretreated with platinum [133].

#### Cabazitaxel-Cancers of the breast and female genital organs

Cabazitaxel (DB06772) is used for the treatment of hormone-refractory metastatic prostate cancer patients pretreated with docetaxel [134]. Moreover, Villanueva *et al.* reported that the combination of cabazitaxel+capecitabine was active in patients with MBC [135].

## Conclusions

In this study, an order-prediction model for drugs and their indications was built using the chemical-chemical interaction information extracted from STITCH. The outstanding performance of our model implicated that the model was feasible for drug-indication prediction, *i.e.* it was more likely that interactive chemicals would treat the same cancers than non-interactive ones. Moreover, it was demonstrated that most of the ‘wrong’ predictions might actually right, which may help reposition drugs to their new indications according to the prediction results.

## Supporting Information

Table S1 List of 68 drugs retrieved from KEGG and cancers they can treat.
(PDF)

Table S2 List of 44 drugs extracted from DrugBank and cancers they can treat.
(PDF)

Table S3 List of drugs with ‘wrong’ 1^st^ order prediction.
(PDF)

## References

[1] Jemal A, Bray F (2011) Center MM, Ferlay J, Ward E, et al (2011) Global cancer statistics. CA Cancer J Clin 61: 69–90. 21296855 10.3322/caac.20107

[2] Walters WP, Green J, Weiss JR, Murcko MA (2011) What do medicinal chemists actually make? A 50-year retrospective. J Med Chem 54: 6405–6416. 21755928 10.1021/jm200504p

[3] Hu L, Huang T, Liu XJ, Cai YD (2011) Predicting protein phenotypes based on protein-protein interaction network. PLoS One 6: e17668. 21423698 10.1371/journal.pone.0017668 PMC3053377

[4] Hu L, Huang T, Shi X, Lu WC, Cai YD, et al (2011) Predicting functions of proteins in mouse based on weighted protein-protein interaction network and protein hybrid properties. PLoS One 6: e14556. 21283518 10.1371/journal.pone.0014556 PMC3023709

[5] Gao P, Wang QP, Chen L, Huang T (2012) Prediction of human genes’ regulatory functions based on proteinprotein interaction network. Protein Pept Lett 19: 910–916. 22486617 10.2174/092986612802084528

[6] Hu LL, Chen C, Huang T, Cai YD, Chou KC (2011) Predicting biological functions of compounds based on chemical-chemical interactions. PLoS One 6: e29491. 22220213 10.1371/journal.pone.0029491 PMC3248422

[7] Chen L, Zeng WM, Cai YD, Feng KY, Chou KC (2012) Predicting Anatomical Therapeutic Chemical (ATC) Classification of Drugs by Integrating Chemical-Chemical Interactions and Similarities. PLoS ONE 7: e35254. 22514724 10.1371/journal.pone.0035254 PMC3325992

[8] Chen L, Lu J, Zhang J, Feng K-R, Zheng M-Y, et al (2013) Predicting Chemical Toxicity Effects Based on Chemical-Chemical Interactions. PLoS ONE 8: e56517. 23457578 10.1371/journal.pone.0056517 PMC3574107

[9] Kuhn M, von Mering C, Campillos M, Jensen LJ, Bork P (2008) STITCH: interaction networks of chemicals and proteins. Nucleic Acids Res 36: D684–688. 18084021 10.1093/nar/gkm795 PMC2238848

[10] Kuhn M, Szklarczyk D, Franceschini A, Campillos M, von Mering C, et al (2010) STITCH 2: an interaction network database for small molecules and proteins. Nucleic Acids Res 38: D552–556. 19897548 10.1093/nar/gkp937 PMC2808890

[11] Kanehisa M, Goto S (2000) KEGG: Kyoto encyclopedia of genes and genomes. Nucleic Acids Research 28: 27–30. 10592173 10.1093/nar/28.1.27 PMC102409

[12] Wishart DS, Knox C, Guo AC, Shrivastava S, Hassanali M, et al (2006) DrugBank: a comprehensive resource for in silico drug discovery and exploration. Nucleic acids research 34: D668–D672. 16381955 10.1093/nar/gkj067 PMC1347430

[13] Ashburn TT, Thor KB (2004) Drug repositioning: identifying and developing new uses for existing drugs. Nat Rev Drug Discov 3: 673–683. 15286734 10.1038/nrd1468

[14] Boguski MS, Mandl KD, Sukhatme VP (2009) Drug discovery. Repurposing with a difference. Science 324: 1394–1395. 19520944 10.1126/science.1169920

[15] Iorio F, Bosotti R, Scacheri E, Belcastro V, Mithbaokar P, et al (2010) Discovery of drug mode of action and drug repositioning from transcriptional responses. Proc Natl Acad Sci U S A 107: 14621–14626. 20679242 10.1073/pnas.1000138107 PMC2930479

[16] Dudley JT, Sirota M, Shenoy M, Pai RK, Roedder S, et al (2011) Computational repositioning of the anticonvulsant topiramate for inflammatory bowel disease. Sci Transl Med 3: 96ra76. 10.1126/scitranslmed.3002648 PMC3479650 21849664

[17] Sirota M, Dudley JT, Kim J, Chiang AP, Morgan AA, et al (2011) Discovery and preclinical validation of drug indications using compendia of public gene expression data. Sci Transl Med 3: 96ra77. 10.1126/scitranslmed.3001318 PMC3502016 21849665

[18] Cheng F, Liu C, Jiang J, Lu W, Li W, et al (2012) Prediction of drug-target interactions and drug repositioning via network-based inference. PLoS Comput Biol 8: e1002503. 22589709 10.1371/journal.pcbi.1002503 PMC3349722

[19] Du P, Li T, Wang X (2011) Recent progress in predicting protein sub-subcellular locations. Expert Review of Proteomics 8: 391–404. 21679119 10.1586/epr.11.20

[20] Gao YF, Chen L, Cai YD, Feng KY, Huang T, et al (2012) Predicting Metabolic Pathways of Small Molecules and Enzymes Based on Interaction Information of Chemicals and Proteins. PLoS ONE 7: e45944. 23029334 10.1371/journal.pone.0045944 PMC3448724

[21] Sharan R, Ulitsky I, Shamir R (2007) Network-based prediction of protein function. Molecular systems biology 3: 88. 17353930 10.1038/msb4100129 PMC1847944

[22] Ng KL, Ciou JS, Huang CH (2010) Prediction of protein functions based on function-function correlation relations. Computers in Biology and Medicine 40: 300–305. 20089249 10.1016/j.compbiomed.2010.01.001

[23] AMPAC, Semichem, Inc.: Shawnee, KS 66216.

[24] COmprehensive DEscriptors for Structural and Statistical Analysis (CODESSA), Semichem, Inc.: Shawnee, KS 66216.

[25] Chen L, Zeng W-M, Cai Y-D, Huang T (2013) Prediction of Metabolic Pathway Using Graph Property, Chemical Functional Group and Chemical Structural Set. Current Bioinformatics 8: 200–207.

[26] Georgiou D, Karakasidis T, Nieto J, Torres A (2009) Use of fuzzy clustering technique and matrices to classify amino acids and its impact to Chou’s pseudo amino acid composition. Journal of theoretical biology 257: 17–26. 19056401 10.1016/j.jtbi.2008.11.003

[27] Ramani RG, Jacob SG (2013) Prediction of P53 Mutants (Multiple Sites) Transcriptional Activity Based on Structural (2D&3D) Properties. PLoS ONE 8: e55401. 23468845 10.1371/journal.pone.0055401 PMC3572112

[28] Matsuta Y, Ito M, Tohsato Y (2013) ECOH: An Enzyme Commission number predictor using mutual information and a support vector machine. Bioinformatics 29: 365–372. 23220570 10.1093/bioinformatics/bts700

[29] Huang T, Chen L, Cai Y, Chou C (2011) Classification and Analysis of Regulatory Pathways Using Graph Property, Biochemical and Physicochemical Property, and Functional Property. PLoS ONE 6: e25297. 21980418 10.1371/journal.pone.0025297 PMC3182212

[30] Deng M, Zhang K, Mehta S, Chen T, Sun F (2003) Prediction of Protein Function Using Protein-protein Interaction Data. Journal of Computational Biology 10: 947–960. 14980019 10.1089/106652703322756168

[31] Chua HN, Sung WK, Wong L (2006) Exploiting indirect neighbours and topological weight to predict protein function from protein-protein interactions. Bioinformatics 22: 1623–1630. 16632496 10.1093/bioinformatics/btl145

[32] http://pubs.acs.org/cen/coverstory/83/8325/8325cisplatin.html. (accessed March 26, 2013).

[33] http://www.cisplatin.org/. (accessed March 26, 2013).

[34] Wheate NJ, Walker S, Craig GE, Oun R (2010) The status of platinum anticancer drugs in the clinic and in clinical trials. Dalton Trans 39: 8113–8127. 20593091 10.1039/c0dt00292e

[35] Prasad SB, Giri A (1994) Antitumor effect of cisplatin against murine ascites Dalton’s lymphoma. Indian J Exp Biol 32: 155–162. 8070834

[36] http://www.drugbank.ca/drugs/DB01181. (accessed March 27, 2013).

[37] Yamaguchi M, Suzuki R, Kwong YL, Kim WS, Hasegawa Y, et al (2008) Phase I study of dexamethasone, methotrexate, ifosfamide, L-asparaginase, and etoposide (SMILE) chemotherapy for advanced-stage, relapsed or refractory extranodal natural killer (NK)/T-cell lymphoma and leukemia. Cancer Sci 99: 1016–1020. 18294294 10.1111/j.1349-7006.2008.00768.x PMC11158592

[38] Yamaguchi M, Kwong YL, Kim WS, Maeda Y, Hashimoto C, et al (2011) Phase II study of SMILE chemotherapy for newly diagnosed stage IV, relapsed, or refractory extranodal natural killer (NK)/T-cell lymphoma, nasal type: the NK-Cell Tumor Study Group study. J Clin Oncol 29: 4410–4416. 21990393 10.1200/JCO.2011.35.6287

[39] http://www.drugbank.ca/drugs/DB01206. (accessed March 27, 2013).

[40] Moore AS, London CA, Wood CA, Williams LE, Cotter SM, et al (1999) Lomustine (CCNU) for the treatment of resistant lymphoma in dogs. J Vet Intern Med 13: 395–398. 10499719 10.1892/0891-6640(1999)013<0395:lfttor>2.3.co;2

[41] Saba CF, Thamm DH, Vail DM (2007) Combination chemotherapy with L-asparaginase, lomustine, and prednisone for relapsed or refractory canine lymphoma. J Vet Intern Med 21: 127–132. 17338160 10.1892/0891-6640(2007)21[127:ccwlla]2.0.co;2

[42] Flory AB, Rassnick KM, Al-Sarraf R, Bailey DB, Balkman CE, et al (2008) Combination of CCNU and DTIC chemotherapy for treatment of resistant lymphoma in dogs. J Vet Intern Med 22: 164–171. 18289305 10.1111/j.1939-1676.2007.0005.x

[43] Williams LE, Rassnick KM, Power HT, Lana SE, Morrison-Collister KE, et al (2006) CCNU in the treatment of canine epitheliotropic lymphoma. J Vet Intern Med 20: 136–143. 16496933 10.1892/0891-6640(2006)20[136:cittoc]2.0.co;2

[44] Heading KL, Brockley LK, Bennett PF (2011) CCNU (lomustine) toxicity in dogs: a retrospective study (2002–07). Aust Vet J 89: 109–116. 10.1111/j.1751-0813.2011.00690.x 21418164

[45] Hahner S, Fassnacht M (2005) Mitotane for adrenocortical carcinoma treatment. Curr Opin Investig Drugs 6: 386–394. 15898346

[46] Luton J-P, Cerdas S, Billaud L, Thomas G, Guilhaume B, et al (1990) Clinical Features of Adrenocortical Carcinoma, Prognostic Factors, and the Effect of Mitotane Therapy. New England Journal of Medicine 322: 1195–1201. 2325710 10.1056/NEJM199004263221705

[47] Jarabak J, Rice K (1981) Metastatic adrenal cortical carcinoma. Prolonged regression with mitotane therapy. JAMA 246: 1706–1707. 7277651

[48] Terzolo M, Angeli A, Fassnacht M, Daffara F, Tauchmanova L, et al (2007) Adjuvant mitotane treatment for adrenocortical carcinoma. N Engl J Med 356: 2372–2380. 17554118 10.1056/NEJMoa063360

[49] Chortis V, Taylor AE, Schneider P, Tomlinson JW, Hughes BA, et al (2013) Mitotane therapy in adrenocortical cancer induces CYP3A4 and inhibits 5alpha-reductase, explaining the need for personalized glucocorticoid and androgen replacement. J Clin Endocrinol Metab 98: 161–171. 23162091 10.1210/jc.2012-2851

[50] Swaffar DS, Horstman MG, Jaw JY, Thrall BD, Meadows GG, et al (1989) Methylazoxyprocarbazine, the active metabolite responsible for the anticancer activity of procarbazine against L1210 leukemia. Cancer Res 49: 2442–2447. 2706632

[51] http://monographs.iarc.fr/ENG/Monographs/vol100A/mono100A-12.pdf. (accessed March 27, 2013).

[52] Halbsguth TV, Nogova L, Mueller H, Sieniawski M, Eichenauer DA, et al (2010) Phase 2 study of BACOPP (bleomycin, adriamycin, cyclophosphamide, vincristine, procarbazine, and prednisone) in older patients with Hodgkin lymphoma: a report from the German Hodgkin Study Group (GHSG). Blood 116: 2026–2032. 20551376 10.1182/blood-2009-11-253211

[53] http://www.drugbank.ca/drugs/DB00853. (accessed March 27, 2013).

[54] Reni M, Zaja F, Mason W, Perry J, Mazza E, et al (2007) Temozolomide as salvage treatment in primary brain lymphomas. Br J Cancer 96: 864–867. 17325700 10.1038/sj.bjc.6603660 PMC2360092

[55] http://www.drugbank.ca/drugs/DB04572. (accessed March 28, 2013).

[56] Majolino I, Davoli M, Carnevalli E, Locasciulli A, Di Bartolomeo P, et al (2007) Reduced intensity conditioning with thiotepa, fludarabine, and melphalan is effective in advanced multiple myeloma. Leuk Lymphoma 48: 759–766. 17454635 10.1080/10428190601186150

[57] Kolb EA, Steinherz PG (2003) A new multidrug reinduction protocol with topotecan, vinorelbine, thiotepa, dexamethasone, and gemcitabine for relapsed or refractory acute leukemia. Leukemia 17: 1967–1972. 14513046 10.1038/sj.leu.2403097

[58] http://www.drugbank.ca/drugs/DB00322. (accessed March 29, 2013).

[59] Hohn DC, Stagg RJ, Friedman MA, Hannigan JF Jr, Rayner A, et al (1989) A randomized trial of continuous intravenous versus hepatic intraarterial floxuridine in patients with colorectal cancer metastatic to the liver: the Northern California Oncology Group trial. J Clin Oncol 7: 1646–1654. 2530317 10.1200/JCO.1989.7.11.1646

[60] Gopal AK, Press OW, Shustov AR, Petersdorf SH, Gooley TA, et al (2010) Efficacy and safety of gemcitabine, carboplatin, dexamethasone, and rituximab in patients with relapsed/refractory lymphoma: a prospective multi-center phase II study by the Puget Sound Oncology Consortium. Leuk Lymphoma 51: 1523–1529. 20578815 10.3109/10428194.2010.491137 PMC3018339

[61] Moskowitz CH, Bertino JR, Glassman JR, Hedrick EE, Hunte S, et al (1999) Ifosfamide, carboplatin, and etoposide: a highly effective cytoreduction and peripheral-blood progenitor-cell mobilization regimen for transplant-eligible patients with non-Hodgkin’s lymphoma. J Clin Oncol 17: 3776–3785. 10577849 10.1200/JCO.1999.17.12.3776

[62] http://www.drugbank.ca/drugs/DB00445. (accessed March 30, 2013).

[63] Rummel MJ, Kafer G, Pfreundschuh M, Jager E, Reinhardt U, et al (1999) Fludarabine and epirubicin in the treatment of chronic lymphocytic leukaemia: a German multicenter phase II study. Ann Oncol 10: 183–188. 10093687 10.1023/a:1008312432416

[64] http://www.drugbank.ca/drugs/DB00441. (accessed March 30,2013).

[65] http://www.drugbank.ca/drugs/DB00361. (accessed March 30, 2013).

[66] Aapro M, Finek J (2012) Oral vinorelbine in metastatic breast cancer: a review of current clinical trial results. Cancer Treat Rev 38: 120–126. 21742438 10.1016/j.ctrv.2011.05.005

[67] Vogel C, O’Rourke M, Winer E, Hochster H, Chang A, et al (1999) Vinorelbine as first-line chemotherapy for advanced breast cancer in women 60 years of age or older. Ann Oncol 10: 397–402. 10370781 10.1023/a:1008364222793

[68] http://www.drugbank.ca/drugs/DB00762. (accessed March 30, 2013).

[69] Perez EA, Hillman DW, Mailliard JA, Ingle JN, Ryan JM, et al (2004) Randomized phase II study of two irinotecan schedules for patients with metastatic breast cancer refractory to an anthracycline, a taxane, or both. J Clin Oncol 22: 2849–2855. 15254052 10.1200/JCO.2004.10.047

[70] Hayashi H, Tsurutani J, Satoh T, Masuda N, Okamoto W, et al (2013) Phase II study of bi-weekly irinotecan for patients with previously treated HER2-negative metastatic breast cancer: KMBOG0610B. Breast Cancer 20: 131–136. 22124996 10.1007/s12282-011-0316-z

[71] Stathopoulos GP, Tsavdaridis D, Malamos NA, Rigatos SK, Kosmas C, et al (2005) Irinotecan combined with docetaxel in pre-treated metastatic breast cancer patients: a phase II study. Cancer Chemother Pharmacol 56: 487–491. 15868147 10.1007/s00280-005-1006-3

[72] Blum JL (1999) Xeloda in the treatment of metastatic breast cancer. Oncology 57 Suppl 116–20. 10436412 10.1159/000055264

[73] http://www.accessdata.fda.gov/drugsatfda_docs/label/2000/20896lbl.pdf. (accessed March 30, 2013).

[74] Ershler WB (2006) Capecitabine monotherapy: safe and effective treatment for metastatic breast cancer. Oncologist 11: 325–335. 16614228 10.1634/theoncologist.11-4-325

[75] http://www.drugbank.ca/drugs/DB00317. (accessed March 30, 2013).

[76] Leibl S, Moinfar F (2005) Metaplastic breast carcinomas are negative for Her-2 but frequently express EGFR (Her-1): potential relevance to adjuvant treatment with EGFR tyrosine kinase inhibitors? J Clin Pathol 58: 700–704. 15976335 10.1136/jcp.2004.025163 PMC1770725

[77] http://www.drugbank.ca/drugs/DB00398. (accessed March 30, 2013).

[78] Blumenschein GR Jr, Gatzemeier U, Fossella F, Stewart DJ, Cupit L, et al (2009) Phase II, multicenter, uncontrolled trial of single-agent sorafenib in patients with relapsed or refractory, advanced non-small-cell lung cancer. J Clin Oncol 27: 4274–4280. 19652055 10.1200/JCO.2009.22.0541

[79] http://www.drugbank.ca/drugs/DB01229. (accessed March 30, 2013).

[80] Hensing TA, Peterman AH, Schell MJ, Lee JH, Socinski MA (2003) The impact of age on toxicity, response rate, quality of life, and survival in patients with advanced, Stage IIIB or IV nonsmall cell lung carcinoma treated with carboplatin and paclitaxel. Cancer 98: 779–788. 12910523 10.1002/cncr.11548

[81] http://www.drugbank.ca/drugs/DB00851. (accessed March 31, 2013).

[82] Baker TR, Piver MS, Caglar H, Piedmonte M (1991) Prospective trial of cisplatin, adriamycin, and dacarbazine in metastatic mixed mesodermal sarcomas of the uterus and ovary. Am J Clin Oncol 14: 246–250. 2031513 10.1097/00000421-199106000-00012

[83] http://www.drugbank.ca/drugs/DB01268. (accessed March 31, 2013).

[84] Burstein HJ, Elias AD, Rugo HS, Cobleigh MA, Wolff AC, et al (2008) Phase II study of sunitinib malate, an oral multitargeted tyrosine kinase inhibitor, in patients with metastatic breast cancer previously treated with an anthracycline and a taxane. J Clin Oncol 26: 1810–1816. 18347007 10.1200/JCO.2007.14.5375

[85] Kozloff M, Chuang E, Starr A, Gowland PA, Cataruozolo PE, et al (2010) An exploratory study of sunitinib plus paclitaxel as first-line treatment for patients with advanced breast cancer. Ann Oncol 21: 1436–1441. 20032126 10.1093/annonc/mdp565 PMC2890319

[86] http://www.drugbank.ca/drugs/DB00499. (accessed August 1, 2013).

[87] Dimonaco M, Brignardello E, Leonardi L, Gatto V, Gallo M, et al (1993) The antiandrogen flutamide inhibits growth of mcf-7 human breast-cancer cell-line. Int J Oncol 2: 653–656. 21573607 10.3892/ijo.2.4.653

[88] http://www.drugbank.ca/drugs/DB00650. (accessed August 21, 2013).

[89] Repetto L, Miglietta L, Gardin G, Lanfranco C, Naso C, et al (1994) Phase II study of weekly mitoxantrone, 5-fluorouracil, and leucovorin in metastatic breast cancer. Breast Cancer Res Treat 30: 133–137. 7949211 10.1007/BF00666056

[90] Rivera E, Chang JC, Semiglazov V, Gorbunova V, Manikhas A, et al. (2012) Eniluracil +5-fluorouracil+leucovorin (EFL) vs. capecitabine phase 2 trial for metastatic breast cancer. Thirty-Fifth Annual CTRC-AACR San Antonio Breast Cancer Symposium. San Antonio, TX. pp. Supplement 3.

[91] Hackshaw A, Baum M, Fornander T, Nordenskjold B, Nicolucci A, et al (2009) Long-term effectiveness of adjuvant goserelin in premenopausal women with early breast cancer. J Natl Cancer Inst 101: 341–349. 19244174 10.1093/jnci/djn498 PMC2650713

[92] http://www.drugbank.ca/drugs/DB00544. (accessed August 1, 2013).

[93] Takeno A, Tamura S, Miki H, Ono H, Uchiyama C, et al (2010) [Successful treatment of advanced esophageal cancer with lymph node metastases by docetaxel, cisplatin and 5-FU followed by salvage lymphadenectomy–a case report]. Gan To Kagaku Ryoho 37: 2382–2384. 21224580

[94] http://www.drugbank.ca/drugs/DB00970. (accessed March 26, 2013).

[95] James DH Jr, Hustu O, Wrenn EL Jr, Johnson WW (1966) Childhood malignant tumors. Concurrent chemotherapy with dactinomycin and vincristine sulfate. JAMA 197: 1043–1045. 4288287 10.1001/jama.197.12.1043

[96] http://www.drugbank.ca/drugs/DB00305. (accessed March 28, 2013).

[97] Yu CS, Chiu SI, Ng CS, Chan HH, Tse RK (2008) Localized conjunctival mucosa-associated lymphoid tissue (MALT) lymphoma is amenable to local chemotherapy. Int Ophthalmol 28: 51–54. 17589808 10.1007/s10792-007-9102-5

[98] Sartorelli AC, Booth BA (1965) The synergistic anti-neoplastic activity of combinations of mitomycins with either 6-thioguanine or 5-fluorouracil. Cancer Res 25: 1393–1400. 5861069

[99] Imai R, Morimoto M (1983) Comparative antitumor activities of 7-N-(p-hydroxyphenyl)mitomycin C (M-83) and mitomycin C. J Antibiot (Tokyo). 36: 559–565. 10.7164/antibiotics.36.559 6409871

[100] http://www.drugbank.ca/drugs/DB00773. (accessed March 31, 2013).

[101] Poplin EA, Liu PY, Delmore JE, Wilczynski S, Moore DF Jr, et al (1999) Phase II trial of oral etoposide in recurrent or refractory endometrial adenocarcinoma: a southwest oncology group study. Gynecol Oncol 74: 432–435. 10479505 10.1006/gyno.1999.5461

[102] Kanazawa K, Moromizato H (1996) [Etoposide (VP-16) in gynecologic malignancy]. Gan To Kagaku Ryoho 23: 1925–1928. 8978799

[103] Kredentser DC (1991) Etoposide (VP-16), ifosfamide/mesna, and cisplatin chemotherapy for advanced and recurrent carcinoma of the cervix. Gynecol Oncol 43: 145–148. 1743556 10.1016/0090-8258(91)90061-9

[104] http://www.drugbank.ca/drugs/DB00255. (accessed in November 7, 2013).

[105] Peethambaram PP, Ingle JN, Suman VJ, Hartmann LC, Loprinzi CL (1999) Randomized trial of diethylstilbestrol vs. tamoxifen in postmenopausal women with metastatic breast cancer. An updated analysis. Breast Cancer Res Treat 54: 117–122. 10424402 10.1023/a:1006185805079

[106] http://www.drugbank.ca/drugs/DB00290. (accessed in November 7, 2013).

[107] Takeuchi K (1975) A clinical trial of intravenous bleomycin in the treatment of brain tumors. Int J Clin Pharmacol Biopharm 12: 419–426. 54345

[108] Agerholm-Larsen B, Iversen HK, Ibsen P, Moller JM, Mahmood F, et al (2011) Preclinical validation of electrochemotherapy as an effective treatment for brain tumors. Cancer Res 71: 3753–3762. 21507935 10.1158/0008-5472.CAN-11-0451

[109] http://www.drugbank.ca/drugs/DB00307. (accessed in November 8, 2013).

[110] Wang Y, Zhang Z, Yao R, Jia D, Wang D, et al (2006) Prevention of lung cancer progression by bexarotene in mouse models. Oncogene 25: 1320–1329. 16247446 10.1038/sj.onc.1209180

[111] Hermann TW, Yen WC, Tooker P, Fan B, Roegner K, et al (2005) The retinoid X receptor agonist bexarotene (Targretin) synergistically enhances the growth inhibitory activity of cytotoxic drugs in non-small cell lung cancer cells. Lung Cancer 50: 9–18. 15993980 10.1016/j.lungcan.2005.05.008

[112] http://www.drugbank.ca/drugs/DB00380. (accessed in November 8, 2013).

[113] Galetta F, Franzoni F, Cervetti G, Cecconi N, Carpi A, et al (2005) Effect of epirubicin-based chemotherapy and dexrazoxane supplementation on QT dispersion in non-Hodgkin lymphoma patients. Biomed Pharmacother 59: 541–544. 16325366 10.1016/j.biopha.2004.12.003

[114] Lipshultz SE, Rifai N, Dalton VM, Levy DE, Silverman LB, et al (2004) The Effect of Dexrazoxane on Myocardial Injury in Doxorubicin-Treated Children with Acute Lymphoblastic Leukemia. New England Journal of Medicine 351: 145–153. 15247354 10.1056/NEJMoa035153

[115] Vrooman LM, Neuberg DS, Stevenson KE, Asselin BL, Athale UH, et al (2011) The low incidence of secondary acute myelogenous leukaemia in children and adolescents treated with dexrazoxane for acute lymphoblastic leukaemia: a report from the Dana-Farber Cancer Institute ALL Consortium. Eur J Cancer 47: 1373–1379. 21514146 10.1016/j.ejca.2011.03.022 PMC3736806

[116] http://www.drugbank.ca/drugs/DB00385. (accessed in November 10, 2013).

[117] Schwartz HS, Kanter PM (1981) DNA damage by anthracycline drugs in human leukemia cells. Cancer Lett 13: 309–313. 6946856 10.1016/0304-3835(81)90059-8

[118] Andersen SM, Rosada C, Dagnaes-Hansen F, Laugesen IG, de Darko E, et al (2010) Topical application of valrubicin has a beneficial effect on developing skin tumors. Carcinogenesis 31: 1483–1490. 20554745 10.1093/carcin/bgq122

[119] http://www.drugbank.ca/drugs/DB00399. (accessed in November 8, 2013).

[120] Steinman RA, Brufsky AM, Oesterreich S (2012) Zoledronic acid effectiveness against breast cancer metastases - a role for estrogen in the microenvironment? Breast Cancer Res 14: 213. 23014660 10.1186/bcr3223 PMC4053096

[121] http://www.drugbank.ca/drugs/DB00642. (accessed in November 8, 2013).

[122] Bajetta E, Celio L, Buzzoni R, Ferrari L, Marchiano A, et al (2003) Phase II study of pemetrexed disodium (Alimta) administered with oral folic acid in patients with advanced gastric cancer. Ann Oncol 14: 1543–1548. 14504056 10.1093/annonc/mdg406

[123] http://www.drugbank.ca/drugs/DB01185. (accessed in November 10, 2013).

[124] Bai GZ, Hara H, Nagai K (1984) Influence of fluoxymesterone on in vitro erythropoiesis affected by leukemic cells. Exp Hematol 12: 171–176. 6584313

[125] http://www.drugbank.ca/downloads. (accessed in November 10, 2013).

[126] Lian F, Li Y, Bhuiyan M, Sarkar FH (1999) p53-independent apoptosis induced by genistein in lung cancer cells. Nutr Cancer 33: 125–131. 10368806 10.1207/S15327914NC330202

[127] http://www.drugbank.ca/drugs/DB02546. (accessed in November 10, 2013).

[128] Pratap J, Akech J, Wixted JJ, Szabo G, Hussain S, et al (2010) The histone deacetylase inhibitor, vorinostat, reduces tumor growth at the metastatic bone site and associated osteolysis, but promotes normal bone loss. Mol Cancer Ther 9: 3210–3220. 21159607 10.1158/1535-7163.MCT-10-0572 PMC3059237

[129] http://www.drugbank.ca/drugs/DB04845. (accessed in November 10, 2013).

[130] Kim YH, Muro K, Yasui H, Chen JS, Ryu MH, et al (2012) A phase II trial of ixabepilone in Asian patients with advanced gastric cancer previously treated with fluoropyrimidine-based chemotherapy. Cancer Chemother Pharmacol 70: 583–590. 22886073 10.1007/s00280-012-1943-6 PMC3456918

[131] Ajani JA, Safran H, Bokemeyer C, Shah MA, Lenz HJ, et al (2006) A multi-center phase II study of BMS-247550 (Ixabepilone) by two schedules in patients with metastatic gastric adenocarcinoma previously treated with a taxane. Invest New Drugs 24: 441–446. 16586011 10.1007/s10637-006-7304-8

[132] http://www.drugbank.ca/drugs/DB05109. (accessed in November 10, 2013).

[133] Massuti B, Cobo M, Camps C, Domine M, Provencio M, et al (2012) Trabectedin in patients with advanced non-small-cell lung cancer (NSCLC) with XPG and/or ERCC1 overexpression and BRCA1 underexpression and pretreated with platinum. Lung Cancer 76: 354–361. 22197612 10.1016/j.lungcan.2011.12.002

[134] http://www.drugbank.ca/drugs/DB06772. (accessed in November 10, 2013).

[135] Villanueva C, Awada A, Campone M, Machiels JP, Besse T, et al (2011) A multicentre dose-escalating study of cabazitaxel (XRP6258) in combination with capecitabine in patients with metastatic breast cancer progressing after anthracycline and taxane treatment: a phase I/II study. Eur J Cancer 47: 1037–1045. 21339064 10.1016/j.ejca.2011.01.001

