# Supplementary material for: Prediction of Cancer Drugs by Chemical-Chemical Interactions
Source: PLoS One. 2014 Feb 3;9(2):e87791. doi: 10.1371/journal.pone.0087791 (PMC3912061; doi:10.1371/journal.pone.0087791)
Supplement: Table S1 — List of 68 drugs retrieved from KEGG and cancers they can treat. (PDF) [file pone.0087791.s001.pdf]

Table S1. 68 drugs investigated in this study and cancers they can treat.

Drug	Cancer
D00214	Cancers of the breast and female genital organs
D00214	Cancers of soft tissues and bone
D00266	Cancers of haematopoietic and lymphoid tissues
D00266	Cancers of the breast and female genital organs
D00208	Cancers of the digestive system
D00208	Cancers of the breast and female genital organs
D05932	Cancers of the digestive system
D05932	Cancers of endocrine organs
D01275	Cancers of the nervous system
D01275	Cancers of the digestive system
D01275	Cancers of haematopoietic and lymphoid tissues
D01275	Cancers of the breast and female genital organs
D01275	Cancers of soft tissues and bone
D01275	Cancers of the urinary system and male genital organs
D01275	Cancers of endocrine organs
D00125	Cancers of the digestive system
D00125	Cancers of haematopoietic and lymphoid tissues
D00125	Cancers of soft tissues and bone
D00125	Cancers of the urinary system and male genital organs
D00125	Cancers of the lung and pleura

D02115	Cancers of haematopoietic and lymphoid tissues
D02115	Cancers of the breast and female genital organs
D02115	Cancers of soft tissues and bone
D02115	Cancers of the urinary system and male genital organs
D01068	Cancers of haematopoietic and lymphoid tissues
D01068	Cancers of the breast and female genital organs
D01068	Cancers of soft tissues and bone
D01068	Cancers of the urinary system and male genital organs
D01068	Cancers of the lung and pleura
D04107	Cancers of the digestive system
D04107	Cancers of haematopoietic and lymphoid tissues
D04107	Cancers of soft tissues and bone
D04107	Cancers of the urinary system and male genital organs
D04107	Cancers of the lung and pleura
D00491	Cancers of the breast and female genital organs
D05333	Cancers of the breast and female genital organs
D00960	Cancers of the breast and female genital organs
D00294	Cancers of endocrine organs
D00586	Cancers of the urinary system and male genital organs
D00341	Cancers of haematopoietic and lymphoid tissues
D00343	Cancers of soft tissues and bone
D00355	Cancers of endocrine organs

D00964	Cancers of the breast and female genital organs
D00989	Cancers of the urinary system and male genital organs
D00363	Cancers of the nervous system
D00420	Cancers of endocrine organs
D05261	Cancers of endocrine organs
D00478	Cancers of the nervous system
D01404	Cancers of haematopoietic and lymphoid tissues
D01441	Cancers of haematopoietic and lymphoid tissues
D06067	Cancers of the nervous system
D00754	Cancers of haematopoietic and lymphoid tissues
D00583	Cancers of the urinary system and male genital organs
D00292	Cancers of haematopoietic and lymphoid tissues
D04197	Cancers of the urinary system and male genital organs
D00473	Cancers of haematopoietic and lymphoid tissues
D01211	Cancers of the digestive system
D03637	Cancers of haematopoietic and lymphoid tissues
D00975	Cancers of haematopoietic and lymphoid tissues
D01370	Cancers of haematopoietic and lymphoid tissues
D01264	Cancers of haematopoietic and lymphoid tissues
D01907	Cancers of haematopoietic and lymphoid tissues
D02214	Cancers of the breast and female genital organs
D01747	Cancers of haematopoietic and lymphoid tissues

D01790	Cancers of the digestive system
D00573	Cancers of the urinary system and male genital organs
D00963	Cancers of the breast and female genital organs
D01935	Cancers of the lung and pleura
D01061	Cancers of the digestive system
D01223	Cancers of the digestive system
D01615	Cancers of haematopoietic and lymphoid tissues
D01510	Cancers of haematopoietic and lymphoid tissues
D01948	Cancers of haematopoietic and lymphoid tissues
D01632	Cancers of haematopoietic and lymphoid tissues
D01977	Cancers of the lung and pleura
D04715	Cancers of the digestive system
D06272	Cancers of the urinary system and male genital organs
D02174	Cancers of haematopoietic and lymphoid tissues
D02250	Cancers of endocrine organs
D00369	Cancers of haematopoietic and lymphoid tissues
D06109	Cancers of haematopoietic and lymphoid tissues
D00966	Cancers of the breast and female genital organs
D06402	Cancers of the urinary system and male genital organs
D03046	Cancers of haematopoietic and lymphoid tissues
D00254	Cancers of the nervous system
D00254	Cancers of haematopoietic and lymphoid tissues

D00275	Cancers of the nervous system
D00275	Cancers of the digestive system
D00275	Cancers of the breast and female genital organs
D00275	Cancers of the urinary system and male genital organs
D00275	Cancers of the lung and pleura
D00287	Cancers of the nervous system
D00287	Cancers of haematopoietic and lymphoid tissues
D00287	Cancers of the breast and female genital organs
D00287	Cancers of soft tissues and bone
D00584	Cancers of the digestive system
D00584	Cancers of the breast and female genital organs
D00584	Cancers of the urinary system and male genital organs
D02197	Cancers of the nervous system
D02197	Cancers of haematopoietic and lymphoid tissues
D02197	Cancers of the breast and female genital organs
D02197	Cancers of soft tissues and bone
D01363	Cancers of the breast and female genital organs
D01363	Cancers of the lung and pleura
D01155	Cancers of the digestive system
D01155	Cancers of the lung and pleura
D02165	Cancers of the breast and female genital organs
D02165	Cancers of the lung and pleura

D00288	Cancers of the nervous system
D00288	Cancers of haematopoietic and lymphoid tissues
D00288	Cancers of soft tissues and bone
