# Supplementary material for: Prediction of Cancer Drugs by Chemical-Chemical Interactions
Source: PLoS One. 2014 Feb 3;9(2):e87791. doi: 10.1371/journal.pone.0087791 (PMC3912061; doi:10.1371/journal.pone.0087791)
Supplement: Table S2 — List of 44 drugs extracted from DrugBank and cancers they can treat. (PDF) [file pone.0087791.s002.pdf]

Table S2. 44 drugs extracted from Drugbank and cancers they can treat.

Drug	Cancer
DB00236	Cancers of haematopoietic and lymphoid tissues
DB00255	Cancers of the urinary system and male genital organs
DB00276	Cancers of haematopoietic and lymphoid tissues
DB00290	Cancers of haematopoietic and lymphoid tissues
DB00290	Cancers of the lung and pleura
DB00293	Cancers of the digestive system
DB00307	Cancers of haematopoietic and lymphoid tissues
DB00309	Cancers of haematopoietic and lymphoid tissues
DB00357	Cancers of the breast and female genital organs
DB00380	Cancers of the breast and female genital organs
DB00385	Cancers of the urinary system and male genital organs
DB00399	Cancers of haematopoietic and lymphoid tissues
DB00399	Cancers of soft tissues and bone
DB00480	Cancers of haematopoietic and lymphoid tissues
DB00481	Cancers of the breast and female genital organs
DB00488	Cancers of the breast and female genital organs
DB00530	Cancers of the digestive system
DB00530	Cancers of the lung and pleura
DB00552	Cancers of haematopoietic and lymphoid tissues
DB00631	Cancers of haematopoietic and lymphoid tissues

DB00642	Cancers of the lung and pleura
DB00665	Cancers of the urinary system and male genital organs
DB00755	Cancers of haematopoietic and lymphoid tissues
DB00765	Cancers of endocrine organs
DB00848	Cancers of the digestive system
DB00888	Cancers of haematopoietic and lymphoid tissues
DB00888	Cancers of the lung and pleura
DB00894	Cancers of the breast and female genital organs
DB01008	Cancers of haematopoietic and lymphoid tissues
DB01033	Cancers of haematopoietic and lymphoid tissues
DB01128	Cancers of the urinary system and male genital organs
DB01143	Cancers of the breast and female genital organs
DB01185	Cancers of the breast and female genital organs
DB01254	Cancers of haematopoietic and lymphoid tissues
DB01259	Cancers of the breast and female genital organs
DB01280	Cancers of haematopoietic and lymphoid tissues
DB01645	Cancers of the urinary system and male genital organs
DB02546	Cancers of haematopoietic and lymphoid tissues
DB03010	Cancers of the nervous system
DB03010	Cancers of the digestive system
DB03010	Cancers of the breast and female genital organs
DB03010	Cancers of the lung and pleura

DB03496	Cancers of the digestive system
DB03496	Cancers of haematopoietic and lymphoid tissues
DB03496	Cancers of the lung and pleura
DB04839	Cancers of the urinary system and male genital organs
DB04845	Cancers of haematopoietic and lymphoid tissues
DB04845	Cancers of the breast and female genital organs
DB04845	Cancers of the urinary system and male genital organs
DB04845	Cancers of the lung and pleura
DB04865	Cancers of haematopoietic and lymphoid tissues
DB04868	Cancers of haematopoietic and lymphoid tissues
DB05109	Cancers of the digestive system
DB05109	Cancers of the breast and female genital organs
DB05109	Cancers of soft tissues and bone
DB05260	Cancers of haematopoietic and lymphoid tissues
DB05812	Cancers of the urinary system and male genital organs
DB06772	Cancers of the urinary system and male genital organs
