# Supplementary material for: Prediction of Cancer Drugs by Chemical-Chemical Interactions
Source: PLoS One. 2014 Feb 3;9(2):e87791. doi: 10.1371/journal.pone.0087791 (PMC3912061; doi:10.1371/journal.pone.0087791)
Supplement: Table S3 — List of drugs with ‘wrong’ 1st order prediction. (PDF) [file pone.0087791.s003.pdf]

Table S3. Drug compounds in three datasets with the ‘wrong’ 1st order prediction, where 26 in $\mathbf{S}_{tr}$, 4 in $\mathbf{S}_{te}$ and 18 in $\mathbf{S}_{ite}$.

(1) 26 drug compounds in $\mathbf{S}_{tr}$ with the ‘wrong’ 1st order prediction

Drug ID	Tag of true cancer	Tag of 1st order prediction
D00275	C_1,C_2,C_4,C_6,C_8	C_3
D00288	C_1,C_3,C_5	C_4
D00294	C_7	C_1
D00343	C_5	C_3
D00363	C_1	C_3
D00420	C_7	C_6
D00478	C_1	C_3
D00573	C_6	C_4
D00583	C_6	C_3
D00584	C_2,C_4,C_6	C_3
D00586	C_6	C_4
D00989	C_6	C_3
D01061	C_2	C_4
D01155	C_2,C_8	C_3
D01211	C_2	C_4
D01223	C_2	C_4
D01363	C_4,C_8	C_3
D01935	C_8	C_4
D01977	C_8	C_4
D02214	C_4	C_3
D02250	C_7	C_6
D04197	C_6	C_2
D05333	C_4	C_8
D06067	C_1	C_3
D06272	C_6	C_8
D06402	C_6	C_4

(2) 4 drug compounds in $\mathbf{S}_{te}$ with the 'wrong' 1st order prediction

Drug ID	Tag of true cancer	Tag of 1st order prediction
D00214	C_4,C_5	C_3
D00208	C_2,C_4	C_3
D05932	C_2,C_7	C_1
D04107	C_2,C_3,C_5,C_6,C_8	C_4

(3) 18 drug compounds in $\mathbf{S}_{ite}$ with the 'wrong' 1st order prediction

Drug ID	Tag of true cancer	Tag of 1st order prediction
DB00255	C_6	C_4
DB00290	C_3,C_8	C_1
DB00307	C_3	C_8
DB00380	C_4	C_3
DB00385	C_6	C_3
DB00399	C_3,C_5	C_4
DB00488	C_4	C_1
DB00642	C_8	C_2
DB00894	C_4	C_6
DB01185	C_4	C_3
DB01645	C_6	C_8
DB02546	C_3	C_6
DB04845	C_3,C_4,C_6,C_8	C_2
DB04868	C_3	C_6
DB05109	C_2,C_4,C_5	C_8
DB05260	C_3	C_8
DB05812	C_6	C_3
DB06772	C_6	C_4
